# Fifth Percentile Cutoff Values for Antipneumococcal Polysaccharide and Anti-*Salmonella typhi* Vi IgG Describe a Normal Polysaccharide Response

**DOI:** 10.3389/fimmu.2017.00546

**Published:** 2017-05-12

**Authors:** Heidi Schaballie, Barbara Bosch, Rik Schrijvers, Marijke Proesmans, Kris De Boeck, Mieke Nelly Boon, François Vermeulen, Natalie Lorent, Doreen Dillaerts, Glynis Frans, Leen Moens, Inge Derdelinckx, Willy Peetermans, Bjørn Kantsø, Charlotte Svaerke Jørgensen, Marie-Paule Emonds, Xavier Bossuyt, Isabelle Meyts

**Affiliations:** ^1^Department of Pediatrics, University Hospitals Leuven, Leuven, Belgium; ^2^St. Giles Laboratory of Infectious Diseases, Rockefeller Branch, The Rockefeller University, New York, NY, USA; ^3^Department Microbiology and Immunology, KU Leuven – University of Leuven, Leuven, Belgium; ^4^Department of Internal Medicine, University Hospitals Leuven, Leuven, Belgium; ^5^Department of Laboratory Medicine, University Hospitals Leuven, Leuven, Belgium; ^6^Virus and Microbiological Special Diagnostics, Statens Serum Institut, Copenhagen, Denmark; ^7^Histocompatibility and Immunogenetic Laboratory, Red Cross Flanders, Mechelen, Belgium

**Keywords:** polysaccharide antibody deficiency, specific antibody deficiency, pneumococcal polysaccharide vaccine, *Salmonella typhi* Vi vaccine, allohemagglutinins

## Abstract

**Background:**

Serotype-specific antibody responses to unconjugated pneumococcal polysaccharide vaccine (PPV) evaluated by a World Health Organization (WHO)-standardized enzyme-linked immunosorbent assay (ELISA) are the gold standard for diagnosis of specific polysaccharide antibody deficiency (SAD). The American Academy of Allergy, Asthma and Immunology (AAAAI) has proposed guidelines to interpret the PPV response measured by ELISA, but these are based on limited evidence. Additionally, ELISA is costly and labor-intensive. Measurement of antibody response to *Salmonella typhi* (*S. typhi*) Vi vaccine and serum allohemagglutinins (AHA) have been suggested as alternatives. However, there are no large cohort studies and cutoff values are lacking.

**Objective:**

To establish cutoff values for antipneumococcal polysaccharide antibody response, anti-*S. typhi* Vi antibody, and AHA.

**Methods:**

One hundred healthy subjects (10–55 years) were vaccinated with PPV and *S. typhi* Vi vaccine. Blood samples were obtained prior to and 3–4 weeks after vaccination. Polysaccharide responses to 3 serotypes were measured by WHO ELISA and to 12 serotypes by an in-house bead-based multiplex assay. Anti-*S. typhi* Vi IgG were measured with a commercial ELISA kit. AHA were measured by agglutination method.

**Results:**

Applying AAAAI criteria, 30% of healthy subjects had a SAD. Using serotype-specific fifth percentile (p5) cutoff values for postvaccination IgG and fold increase pre- over postvaccination, only 4% of subjects had SAD. One-sided 95% prediction intervals for anti-*S. typhi* Vi postvaccination IgG (≥11.2 U/ml) and fold increase (≥2) were established. Eight percent had a response to *S. typhi* Vi vaccine below these cutoffs. AHA titer p5 cutoffs were ½ for anti-B and ¼ for anti-A.

**Conclusion:**

We establish reference cutoff values for interpretation of PPV response measured by bead-based assay, cutoff values for *S. typhi* Vi vaccine responses, and normal values for AHA. For the first time, the intraindividual consistency of all three methods is studied in a large cohort.

## Introduction

Vaccine responses to polysaccharide vaccines are important for the diagnosis and management of patients with suspected primary immunodeficiencies (PID) (1). Several genetically defined PIDs are associated with a specific polysaccharide antibody deficiency (SAD), for example, Wiskott–Aldrich Syndrome, ataxia telangiectasia, 22q11.2 deletion, NEMO deficiency, and autosomal dominant hyper-IgE syndrome (2). Furthermore, responses to polysaccharides can be impaired in patients with IgG subclass deficiency, selective IgM deficiency, selective IgA deficiency, and asplenia (2). A diagnosis of SAD is made when there is an isolated defect of the pneumococcal polysaccharide response with normal responses to protein and conjugate vaccines and normal immunoglobulin levels (3). Patients with SAD suffer from recurrent bacterial respiratory tract infections, such as sinusitis, otitis with chronic otorrhea, bronchitis, or pneumonia. SAD is found in 12–27% of children investigated for recurrent respiratory tract infections (4–8). Patients with increased susceptibility to infections and deficient polysaccharide response can be treated with prophylactic antibiotics, immunoglobulin replacement therapy, or both.

Response to non-conjugated pneumococcal polysaccharide vaccine (PPV), assessed by a World Health Organization (WHO)-standardized Enzyme-Linked Immunosorbent Assay (ELISA), is the gold standard for evaluating T-independent responses (9). Serotype-specific IgG are measured before and after vaccination with PPV. The consensus statement of the American Academy of Allergy, Asthma and Immunology (AAAAI) on diagnostic vaccination defines a normal polysaccharide antibody response in patients above 18 months old as (i) a postvaccination serotype-specific IgG concentration equal to or greater than 1.3 µg/ml and (ii) a twofold increase of the postvaccination concentration when compared to the pre-vaccination concentration (unless pre-vaccination IgG > 4 µg/ml). Children under age 6 years should achieve these cutoffs for at least 50% of serotypes tested, individuals 6 years and older for 70% of tested serotypes. Although these criteria are now widely adopted among PID experts, cutoffs are arbitrary and based on limited evidence due to historical changes and perfection of the specific antibody detection method (radio-immunoassay to ELISA, addition of cell wall polysaccharide, and 22F) (9, 10). Immunological memory for pneumococcal polysaccharides can be found in most individuals following previous infection or nasopharyngeal carriage. Since the introduction of the conjugated pneumococcal vaccine for routine immunization of children, in most industrialized countries, even more individuals have been primed with conjugated polysaccharides (11, 12). Therefore, the measurement of pneumococcal polysaccharide antibodies may no longer be the preferable assessment of the T-independent response (13). Other limitations include cost, workload, and concerns about hyporesponsiveness to polysaccharide vaccines with subsequent administrations (14).

To overcome difficulties with the PPV, two alternative strategies for evaluating the polysaccharide response in patients with suspected PID have been proposed: quantification of *Salmonella typhi* Vi vaccine response or allohemagglutinins (AHA), formerly named isohemagglutinins. Yet, none of these alternatives has been studied against the gold standard in a large control population, and reference values are lacking.

*Salmonella typhi* (*S. typhi*) Vi vaccine (Typhim Vi™, Sanofi Pasteur MSD) is a potential alternative polysaccharide vaccine for evaluating the polysaccharide response in patients with suspected PID. *S. typhi* Vi vaccine contains purified Vi capsular polysaccharide of *S. typhi*, to which the specific antibody response can reliably be measured by ELISA (15, 16). Seroconversion rates were described in two small healthy cohorts [*N* = 26 (15) and *N* = 16 (16)]; the results were not compared to the gold standard pneumococcal antibody assay.

Measurement of AHA is widely adopted as an alternative measurement of polysaccharide responses (9, 12, 17–19). AHA are antibodies reactive to the A or B polysaccharide antigens on erythrocytes. Surprisingly, despite their universal utilization in diagnosis, there were no data on the diagnostic value of AHA for SAD, only anecdotal reports of low or absent AHA in PID. We have recently conducted a retrospective study in patients with suspected PID and found a low diagnostic value of AHA to predict a SAD (20). Data on normal values of AHA in healthy individuals, using current detection methods with differentiation of IgM and IgG, are lacking.

The aim of this study was to establish cutoff values for antipneumococcal polysaccharide, anti-*S. typhi* Vi antibody, and AHA responses.

## Materials and Methods

### Study Design

We conducted a prospective cohort study in 100 healthy volunteers. The study was approved by the Ethics committee of University Hospitals Leuven, Belgium. Volunteers aged 2–55 years were included when they did not meet any of the following exclusion criteria: (i) previous vaccination with an unconjugated polysaccharide salmonella vaccine or pneumococcal vaccine within 5 years prior to study participation, (ii) previous allergic reaction to any vaccine, and (iii) medical history suggestive of PID. After obtaining full informed and written consent, a blood sample was drawn for pre-vaccination serum, allohemagglutinins (AHA), and blood group. Typhim Vi™ (Sanofi Pasteur, Lyon, France) and Pneumovax 23™ (Merck Sharp en Dohme B.V., Haarlem, the Netherlands) vaccines were administered by intramuscular injection at two distinct sites (right and left deltoid muscle). Information on the clinical history and potential previous contact with *S. typhi* was obtained by a medical doctor and noted in a case report form. Three to four weeks after vaccination, a second blood sample was obtained for postvaccination antibody concentrations. Pre- and postvaccination blood was separated by centrifugation and serum was stored at −20°C until simultaneous analysis of specific IgG.

### Antibody Response to PPV

Antipneumococcal polysaccharide IgG against three serotypes that are not in the conjugated vaccine, 8, 9N and 15B, were measured by the third-generation WHO ELISA, incorporating adsorption of samples with cell wall polysaccharide and capsular polysaccharide 22F (20). We have previously demonstrated high precision of the ELISA assay in our laboratory (11, 21). Antibody measurement of specific antipneumococcal IgG antibodies to serotypes 1, 3, 4, 5, 6B, 7F, 9V, 14, 18C, 19A, 19F, and 23F (serotypes included in both 13-valent conjugated pneumococcal vaccine and PPV) was performed at the Statens Serum Institute using an in-house Luminex bead-based assay. This method permits the simultaneous measurement of all 12 serotype-specific IgG in a single well. Briefly, pneumococcal polysaccharides purchased from LGC Standards (American Type Culture Collection, VA, USA) or from SSI Diagnostica (Hilleroed, Denmark) were conjugated to poly-l-lysine and then covalently bound to carboxylated microspheres (Luminex, TX, USA). Serum samples (and the 89SF standard) were pre-adsorped in adsorbance buffer containing CWPS Multi (SSI Diagnostica, Hilleroed, Denmark) and then incubated with the conjugated microspheres, followed by incubation with R-phycoerythrin conjugated anti-human IgG (Jackson ImmunoResearch laboratories, West Grove, PA, USA). Finally, the microspheres were read on a Bio-Plex 200 system (Bio-Rad, Hercules, CA, USA). Data were acquired using Bio-Plex Manager 5.0 (Bio-Rad Hercules, CA, USA). Pneumococcal polysaccharide serum calibrated to the FDA 89SF reference serum was used as a reference (22). Serum IgG concentrations were calculated using a standard curve of median fluorescent intensity (MFI) against expected IgG concentration for FDA 89-SF and converted to micrograms per milliliter. Pre- and postimmunization samples were analyzed on the same plate. Each sample was analyzed in duplicate, and analysis was repeated if the coefficient of variation between duplicates was above 10%.

### Antibody Response to *S. typhi* Vi Vaccine

Specific antibodies to *S. typhi* Vi vaccine were measured using a commercially available ELISA kit (VaccZyme™ Anti-*S. typhi* Vi human IgG EIA kit from The Binding Site Group Ltd., Birmingham, UK). Samples, standards, and quality controls were run in duplicate following the manufacturer instructions. Pre- and postvaccination samples were analyzed on a single plate in a single run. The results were expressed as units per milliliter (range 7.4–600 U/ml). Samples resulting in a value below the lower limit of detection (<7.4 U/ml) were set to 3.7 U/ml. Values above the upper limit of detection (600 U/ml) are reported as 600 U/ml.

### Detection of AHA

Blood group and AHA were determined on pre-vaccination EDTA blood samples from all subjects. Anti-A and anti-B IgG and IgM (AHA) were determined by the immunohematology laboratory of the Red Cross Flanders, using column agglutination technology (Bio-Rad^®^) with LISS Coombs columns and neutral columns, respectively, as previously described (20).

### Statistical Analysis

Statistical calculations were performed using Graphpad Prism version 7.0 for Mac OS X (Graphpad software, La Jolla, CA, USA). Since only low antibody levels are clinically relevant for disease, the lower fifth percentile value (p5) or the one-sided 95% prediction interval (PI) were calculated and used as cutoffs. For antipneumococcal IgG, fold increase of antipneumococcal polysaccharide antibodies and AHA p5 were determined. For anti-*S. typhi* Vi IgG and fold increase of anti-*S. typhi* Vi antibodies, geometric mean and the one-sided 95% prediction interval (PI) were derived from the log-transformed data. The following formula was applied on the log-transformed results: $95\%\text{PI}=\text{mean}-t_{0.95,n-1}\sqrt{\frac{n+1}{n}}\text{SD}$. *t*_0.95,_*_n_*_ − 1_ is the 95% quantile of a Student’s *t* distribution with *n* − 1 degrees of freedom. *N* is the sample size. Geometric mean and 95% PI were then derived by exponentiating the log-derived mean and 95% PI.

## Results

### Study Population

Forty-three males and 57 females were included, aged 10–55 years (median 25 years, IQR 23–30 years). All subjects were of Caucasian descent except for one female of Ethiopian origin. Ten subjects were aged below 19 years, and 6 subjects were aged above 49 years (Figure S1 in Supplementary Material). The median interval between the vaccination and postvaccination blood sample was 24 days (IQR 21–28 days).

None of the subjects had been vaccinated with pneumococcal conjugated vaccine. Five included subjects had previously been vaccinated with *S. typhi* polysaccharide vaccine (≥9 years prior to study participation). None of the subjects noted a known history of *S. typhi* infection. Three subjects had lived in an endemic *S. typhi* area for 1 month or longer (1 month, 3 months, and 6 years).

Two subjects reported one episode of pneumonia in the past 5 years. None declared to suffer from chronic lung disease. Number of upper respiratory tract infections in the last year was reported to be 0 or 1, 2–3, 4–5 or >5 in 72, 24, 3, and 1 subject, respectively. A lifelong history of more than one episode of prolonged otorrhea (>10 days) was reported in three subjects. None of the included subjects had experienced invasive infections. Tooth abscess and appendicitis were mentioned in two and three individuals, respectively.

No serious or severe vaccine-related adverse events were reported. Minor adverse events included fever (*n* = 3), local redness at the site of PPV injection (*n* = 6), local redness at the site of *S. typhi* Vi vaccine injection (*n* = 1), and local tenderness.

### Pneumococcal Polysaccharide Antibody Response

Paired pre- and postvaccination anti-polysaccharide IgG against serotypes 8, 9, and 15B, measured by ELISA, and pre-vaccination IgG levels against fold-increase are shown in Figure S2 in Supplementary Material. Using the AAAAI guidelines for SAD, 3 subjects responded to 0/3 ELISA-tested serotypes, 8 responded to 1/3 serotypes, 32 to 2/3, and 57 to 3/3 serotypes. When a response above the cutoffs for 67% (2/3) of serotypes is accepted as an adequate response, 11/100 healthy subjects would have a SAD (Table 1). When applied to all 15 serotypes, including those assessed by bead-based assay, 30/100 subjects respond to <10/15 serotypes and would have a SAD (Table 1). The median number of serotypes with adequate response was 11 (IQR 9–13).

**Table 1:** **Number of subjects classified with specific polysaccharide antibody deficiency among 100 healthy volunteers for each test method separately (ELISA or bead-based assay) and for all serotypes in total, according to the applied interpretation method: (A) American Academy of Allergy, Asthma and Immunology (AAAAI) criteria (normal response is postvaccination IgG ≥ 1.3 µg/ml and ≥2-fold increase for at least 67% of serotypes) or (B) calculated fifth percentile (p5) cutoffs for postvaccination IgG and fold increase (normal response is postvaccination IgG ≥ serotype-specific p5 value and fold increase ≥ serotype-specific p5 value for at least 67% of serotypes)**.

	ELISA serotypes only (8, 9N, 15B)	Bead-based assay serotypes only (1, 3, 4, 5, 6B, 7 F, 9 V, 14, 18C, 19 A, 19 F, 23 F)	All serotypes
AAAAI criteria applied	11	34	30
p5 cutoffs applied	2	4	4
AAAAI and p5 cutoffs	2	4	4

**Figure 1:**
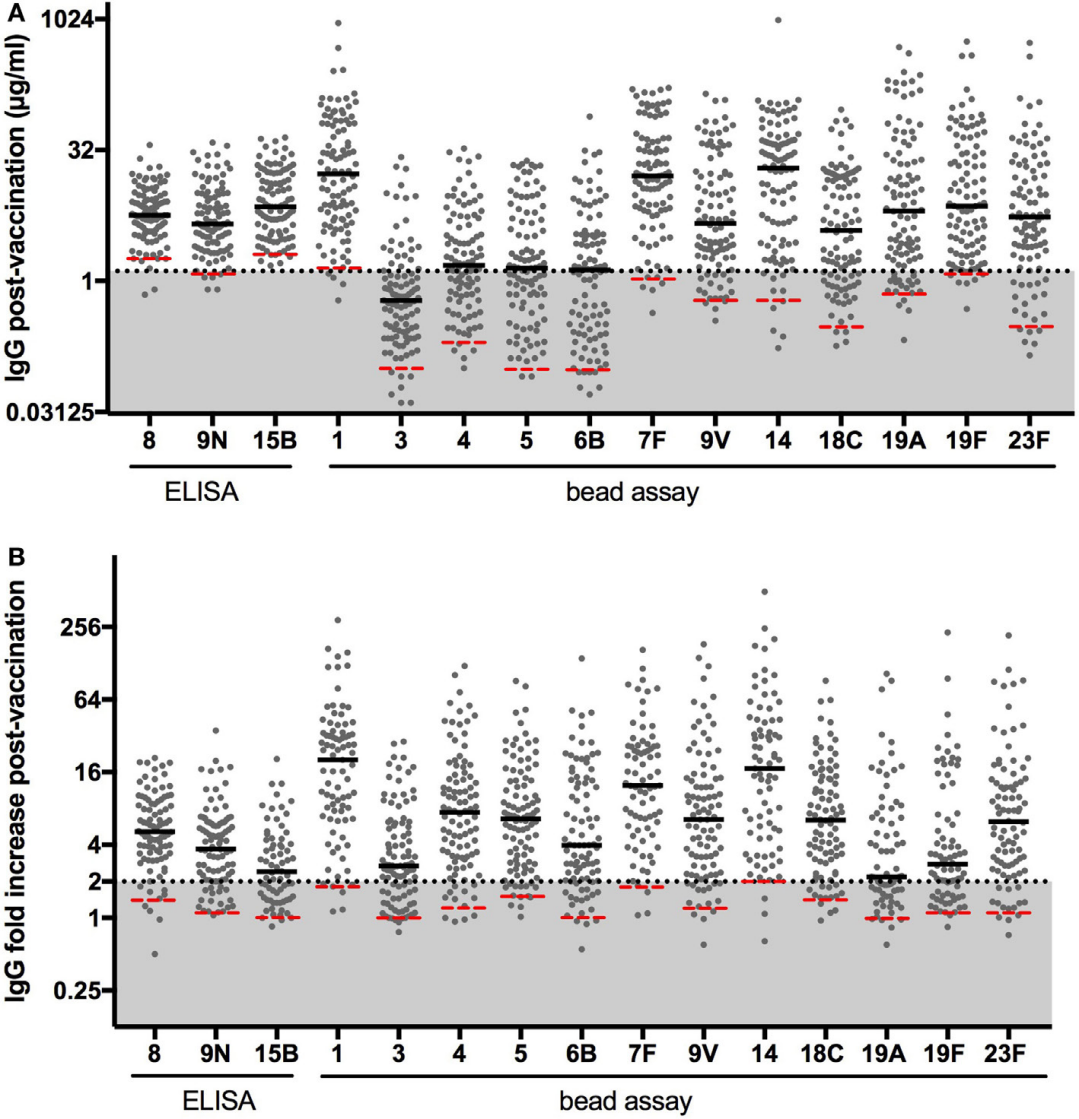
**Polysaccharide-specific IgG levels postvaccination (A) and fold increase (B) of polysaccharide-specific antibody concentration 3–4 weeks after vaccination with pneumococcal polysaccharide vaccine**. Horizontal bars indicate medians; red dashed lines indicate fifth percentile values. The 1.3-µg/ml cutoff and twofold increase cutoff, as advised by the American Academy of Allergy, Asthma and Immunology guidelines, are drawn in dotted lines and area below the cutoff is filled in gray.

As can be seen from Figure 1, a 1.3-µg/ml cutoff for postvaccination IgG and a twofold increase are too high as thresholds for some less immunogenic serotypes, when measured by bead-based assay. We, therefore, determined serotype-specific cutoffs. D’Agostino and Pearson normality test showed that the results were not normally distributed. A normal distribution was found for postvaccination IgG and fold increase for some serotypes when log-transformed, but not for all (log-transformed postvaccination IgG normally distributed for serotypes 8, 9N, 1, 4, 7F, 14, and 23F; log-transformed fold increase normally distributed for serotypes 8, 9N, 15B, 1, 4, 5, 7F, 9V, 14, and 18C). Since only low values for antibody response are of clinical significance, p5 for postvaccination IgG in the cohort was determined to establish cutoff values for the healthy population (Table 2). To determine the fold increase median and p5, subjects with a pre-vaccination IgG level >4 μg/ml were excluded, because fold increase decreases with increasing pre-vaccination titers and a >2-fold increase becomes unlikely above this IgG level (23). Five percent of the healthy volunteers showed no increase in antibody titer for serotypes 15B, 3, 6B, and 19A (Table 2). A p5 of twofold was only found for serotype 14. For all other serotypes, the p5 cutoff for fold increase was between one and two.

**Table 2:** **Serotype-specific IgG and fold increase 3–4 weeks postvaccination with pneumococcal polysaccharide vaccine**.

	Serotype	Postvaccination IgG (μg/ml)				Fold increase			
		Median	Q1	Q3	p5	Median	Q1	Q3	p5
Enzyme-linked immunosorbent assay	8	5.7	3.7	9.7	1.8	5.2	3.4	9.4	1.4
	9N	4.5	2.6	10.1	1.2	3.7	2.1	6.2	1.1
	15B	7.2	3.5	17.2	2.0	2.4	1.4	4.5	1.0
Bead-based assay	1	17.0	5.1	64.7	1.4	20.3	6.9	34.2	1.8
	3	0.6	0.2	1.2	0.1	2.7	1.6	6.1	1.0
	4	1.5	0.7	4.8	0.2	7.4	3.2	15.1	1.2
	5	1.4	0.5	5.2	0.1	6.6	2.8	13.6	1.5
	6B	1.3	0.3	3.4	0.1	4.0	2.1	13.7	1.0
	7F	16.1	6.7	41.8	1.1	12.4	5.6	26.3	1.8
	9V	4.6	1.9	17.1	0.6	6.5	2.5	14.4	1.2
	14	19.8	2.8	41.3	0.6	17.2	4.8	43.3	2.0
	18C	3.8	1.0	15.8	0.3	6.4	3.0	14.9	1.4
	19A	6.4	1.8	29.4	0.7	2.2	1.7	7.3	1.0
	19F	7.3	2.2	33.9	1.2	2.8	1.6	7.2	1.1
	23F	5.4	2.0	20.0	0.3	6.2	2.7	14.0	1.1

*Q1, 25th percentile (quartile 1); Q3, 75th percentile (quartile 3); p5, fifth percentile*.

Next, we redetermined the number of subjects with a SAD, defining a normal response as postvaccination IgG ≥ serotypes-specific p5 and fold increase ≥p5 for at least 67% of serotypes. Again, when pre-vaccination concentration was above 4 µl/ml, no threshold was used for fold increase. Using these criteria, only 2/100 subjects are classified with a SAD when only taking into account the ELISA serotypes and 4/100 subjects when only considering the bead-based assay results (Table 1).

### *S. typhi* Vi Vaccine Antibody Response

Paired pre- and postvaccination specific antibody levels are shown in Figure S3 in Supplementary Material. Pre-vaccination, 73/100 subjects had anti-*S. typhi* Vi IgG below the detection limit of the ELISA kit (7.4 U/ml). Figure S4 in Supplementary Material sets pre-vaccination IgG levels against fold increase. Three subjects had a pre-vaccination anti-*S. typhi* Vi IgG level above 100 U/ml: one had previously been vaccinated and one had been living in Ethiopia for 6 years. Median, p5, geometric mean and one-sided 95% PI for postvaccination anti-*S. typhi* Vi IgG, and fold increase can be found in Table 3. Since the data were log-normal distributed, we propose to use the 95% PI as cutoff for normal values for postvaccination IgG (≥11.2 U/ml) and fold increase (≥2). Sub-analyses in the cohort aged 10–18 and 50–55 years showed comparable 95% PI values. Only 33% of subjects with a pre-vaccination anti-*S. typhi* Vi IgG level above 100 U/ml, were able to mount a twofold increase. When a normal response to *S. typhi* Vi vaccine is defined as postvaccination anti-*S. typhi* Vi IgG ≥11.2 U/ml and a fold increase ≥2 (unless pre-vaccination titer ≥100 U/ml), 8/100 healthy volunteers have an abnormal response to *S. typhi* Vi polysaccharide. Four among these eight with abnormal response had pre-vaccination titer above the detection limit and history of exposure (vaccination) was present in two out of four. Three out of five with history of previous vaccination showed an adequate response, despite detectable anti-*S. typhi* Vi IgG pre-vaccination.

**Table 3:** **Anti-*Salmonella typhi* Vi IgG and fold increase 3–4 weeks postvaccination with *S. typhi* Vi vaccine**.

*n* = 100	Median	IQR	p5	Geometric mean	One-sided ^a^95% PI
Postvaccination anti-*S. typhi* Vi IgG (U/ml)	71.5	36.8–148.7	10.3	72.2	11.2^a^
Fold increase	15	6–24	1	12	2^a^

*IQR, interquartile range; p5, fifth percentile; PI, prediction interval*.

*^a^Proposed normal S. typhi Vi vaccine response*.

### Allohemagglutinins

Two subjects had no AHA because they had blood group AB. Blood group A was found in 42 subjects, blood group B in 11, and blood group O in 45. Therefore, anti-A could be detected in 56 subjects with blood group B or O, and anti-B in 87 subjects with blood group A or O. AHA titers are summarized in Table 4 and Figure S5. When AHA at or above p5 are considered normal, abnormal AHA can be defined as anti-B (IgM or IgG) below ½ or anti-A (IgM or IgG) below ¼. None of the subjects had all AHA below p5. Five subjects had one (*n* = 4) or two (*n* = 1) titers below p5.

**Table 4:** **Allohemagglutinins in 98 healthy subjects with blood group A (*n* = 42), B (*n* = 11), or O (*n* = 45)**.

	Median	IQR	Minimum	Maximum	p5
Anti-B IgG (*n* = 87)	16	8–64	1	2048	2
Anti-B IgM (*n* = 87)	32	8–64	2	2048	2
Anti-A IgG (*n* = 56)	64	20–256	1	2048	4
Anti-B IgM (*n* = 56)	64	16–128	2	512	4

*IQR, interquartile range; p5, fifth percentile*.

### Intraindividual Correlation of Polysaccharide Response Tests

**Figure 2:**
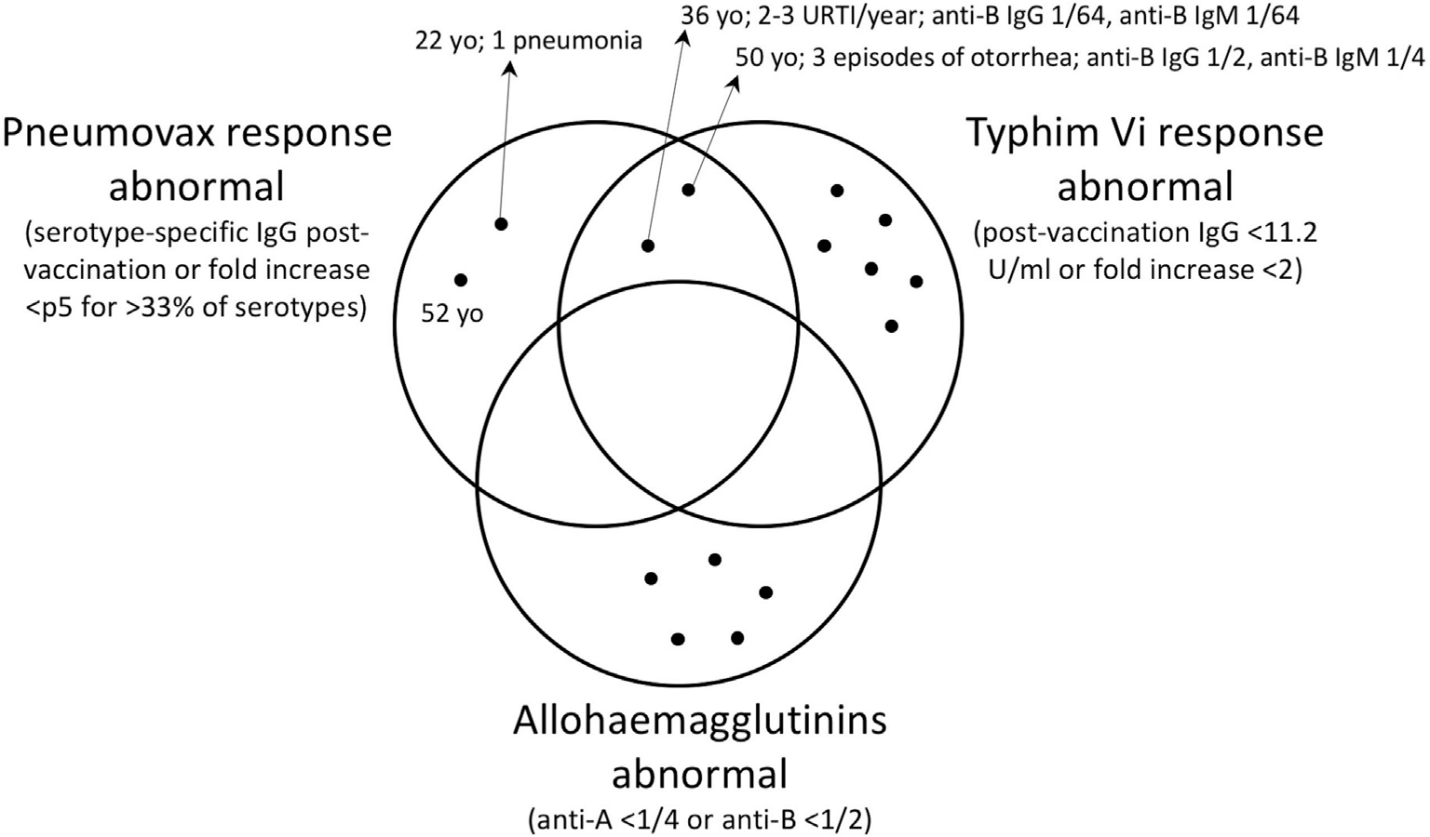
**Venn diagram showing the subjects (black dots) with abnormal antibody response for each detection method among 100 healthy subjects, when the calculated fifth percentile cutoff values are used**. The subjects with abnormal results for more than one test are found in the intersections. Subjects with normal results on all tests are not shown. Where present, the relevant clinical history is indicated by the arrows. URTI, upper respiratory tract infections; yo, years old.

Next, we assessed whether low pneumococcal polysaccharide response corresponded to low Vi polysaccharide response and low response to blood group polysaccharides within one individual. Figure 2 shows the number of individuals with abnormal test results for these three different test methods of polysaccharide response. Detailed results of all 15 subjects with one or more abnormal test result are shown in Table S1 in Supplementary Material. An abnormal test result is defined as a response below the p5 as described above. None of the control subjects had an abnormal response in all three tests. Only two out of eight of controls with a defective *S. typhi* Vi vaccine response had a defect in the response to PPV.

## Discussion

In this study, we established cutoff values for an adequate response to *S. typhi* Vi vaccine and AHA (IgG and IgM) in the largest healthy population reported to date (*n* = 100). To determine the significance of inadequate *S. typhi* Vi vaccine response and low AHA, we compared these polysaccharide responses to the current gold standard: pneumococcal antibody response to PPV.

The WHO ELISA antipneumococcal polysaccharide IgG normal values remain a matter of debate. Applying the AAAAI expert guidelines for SAD diagnosis to the three ELISA-tested serotypes, we found 11% of the cohort had an inadequate response. Applying the AAAAI guidelines to the 15 serotypes (12 bead-based assay tested), we found 30% of the cohort had an inadequate response. In contrast, when the fifth percentile values were used as cutoffs, only 4% had an inadequate response. Although the AAAAI threshold for postvaccination IgG of 1.3 µg/ml was in the range of p5 for the ELISA-tested serotypes, for bead-based assay tested serotypes the p5 was below 1 µg/ml for 9/12 serotypes. Our group has previously suggested using serotype-specific p5 cutoffs for postvaccination IgG for ELISA as well as bead-based assay (21, 24). The AAAAI criteria are derived from ELISA data. Previous studies have shown a good correlation of ELISA with bead-based assay results but the absolute antibody concentrations are not the same. Also, this was not the case for all serotypes (e.g., serotype 3) and correlations were poorer in non-vaccinated subjects and infants (24–28). For fold increase, a p5 of about two is attained for only three bead-assay tested serotypes and for none of the ELISA-tested serotypes. Fifth percentile cutoffs for fold increase have never been published but our findings are consistent with a meta-analysis of older studies and our earlier xMAP study, which showed that a proportion of healthy subjects do not mount a twofold increase, and this proportion is serotype and age dependent (29, 30). Based on these studies and our present findings, we advise to use method- and serotype-specific cutoffs for fold increase as well as postvaccination IgG. Therefore, the proposed p5 cutoffs were used in this study to compare PPV response and *S. typhi* Vi vaccine response within one individual.

We have tested *S. typhi Vi* response in 100 healthy individuals and defined p5 for postvaccination anti-*S. typhi* Vi IgG (<11.2 U/ml) and fold increase (<two-fold) as cutoff for diagnosis of SAD. We, thereby, addressed three major limitations of previous studies describing *S. typhi* Vi antibody responses: limited number of healthy individuals studied (15, 16), use of an in-house hence not widely available ELISA (15), radio-immuno assay (31), or bead-based assay (32) for detection of anti-*S. typhi* Vi antibodies and restricted age groups [in children 2–16 years (33) or 2–5 years (34) old]. A comparison of pneumococcal polysaccharide antibody response and *S. typhi* Vi vaccine response was recently conducted in 16 healthy volunteers, 22 common variable immunodeficiency patients, and 27 hypogammaglobulinemic patients and showed superiority of anti-*S. typhi* Vi vaccine responses to discriminate between groups. However, pneumococcal response was assessed by a commercial PPV-coated ELISA and these ELISAs, measuring a combined IgG response, have limited value to diagnose patients with SAD (13). Using the same commercial ELISA kit for *S. typhi* Vi vaccine, Sánchez-Ramón and colleagues found a minimum postvaccination IgG concentration of 32 and 7.4 U/ml and minimum fold increase of 3.4 and 1 in a healthy and Common Variable Immunodeficiency group, respectively, which shows that our proposed criteria discriminate healthy subjects from SAD subjects (16). Ferry et al. found a greater than threefold increase in 95% of 23 healthy vaccinated individuals, using an in-house ELISA (15).

As expected in an industrialized country, most of the subjects had undetectable anti-*S. typhi* Vi IgG pre-vaccination (73%). This is a major advantage of *S. typhi* Vi vaccine for the evaluation of polysaccharide responsiveness, although it impedes calculation of a fold increase. We have tackled this issue by assigning a value of half the lower detection limit to these subjects. When using the threshold for fold increase as proposed here, the same method will have to be applied. Another difficulty is to define above which pre-vaccination anti-*S. typhi* Vi IgG level this increase should not be expected. A study with PPV has shown a very low likelihood of increase of specific antibody concentrations at given pre-vaccination concentrations. These concentrations are serotype-specific (23). To our knowledge, a similar study has not been conducted for *S. typhi* Vi vaccine. We have arbitrarily chosen 100 U/ml as a threshold for high pre-vaccination antibody concentration. Further studies are needed to determine whether this threshold is correct and needed at all. The inclusion of subjects with a history of *S. typhi* Vi vaccination and subjects who have lived in endemic area has revealed some interesting findings. Although only 6 subjects had a definite encounter with Vi antigen in the past, 27 subjects showed detectable pre-vaccination anti-*S. typhi* Vi antibodies. Half of the subjects with *S. typhi* Vi vaccine response below our proposed criteria had pre-vaccination antibodies, two of them with a history of *S. typhi* Vi vaccination, demonstrating that previous vaccination probably impedes the use of these criteria or this test to assess polysaccharide responsiveness. This hyporesponsiveness, i.e., lower response to subsequent vaccinations with a polysaccharide vaccine, has been described for the pneumococcal and meningococcal polysaccharide vaccines as well (35).

Allohemagglutinins are used by many PID experts and advised by the AAAAI guidelines to assess polysaccharide antibody response, although reference values have been lacking. We have previously found low sensitivity and specificity of AHA titers in the diagnosis of a SAD in 180 subjects with suspected humoral immunodeficiency (20). In the current study, we found a large range of AHA titers in healthy subjects with p5 cutoffs as low as ½ for anti-B and ¼ for anti-A. These thresholds are much lower than the cutoffs currently used (1/8 for both below the age of 3 years and 1/16 above 3 years). We found no association of low AHA with low *S. typhi* Vi vaccine or PPV response. Therefore, these data further question the clinical value of AHA in the diagnosis of SAD. AHA are generated in response to gut bacteria and cross-react with AB blood group antigens (36, 37). Absence of AHA may, therefore, reflect either inability to generate polysaccharide antibodies (in patients) or differences in gut microbiome (in healthy individuals).

This is the first study to compare serotype-specific pneumococcal polysaccharide antibody response with *S. typhi* Vi vaccine response and AHA. We found an overlap of inadequate vaccine response in two subjects. Remarkably, one of these subjects had low AHA and had a history of prolonged otorrhea, a clinical sign that has been shown to be most predictive of SAD in patients with recurrent respiratory tract infections (5). The presence of undiagnosed SAD in this subject is possible. All other subjects with either low PPV response, low *S. typhi* Vi vaccine response, or both, had no or very little (only one pneumonia) clinical signs suggestive of SAD. A partially impaired polysaccharide response may lead to adequate responses to some polysaccharides and an inadequate response to others, without increased susceptibility to infection with encapsulated bacteria. Measuring the response to a single polysaccharide as with the *S. typhi* Vi assay may, therefore, lead to false positive and false negative results. Application of the established cutoff values in patients with suspected antibody deficiency will teach us further whether *S. typhi* Vi vaccine response is a valuable alternative or an additional tool for the diagnosis of SAD. A follow-up study in 100 individuals with suspected humoral immunodeficiency is currently under analysis.

There are also limitations to this study. To cross-validate the two methods to detect antipneumococcal polysaccharide antibodies, ELISA and bead-based assay, the same serotypes should have been tested with both methods. Ideally, only response to non-PCV serotype should be measured, although this is of less importance in this cohort of non-PCV-vaccinated subjects. Additionally, using only three serotypes to define a normal or abnormal response is probably not optimal, especially because of the 50/70% rule on number of serotypes needed with an adequate response.

In conclusion, this is the largest study to date establishing normal values for postvaccination IgG and fold increase of IgG postvaccination with *S. typhi* Vi vaccine, measured with a commercially available ELISA kit. We confirmed that the AAAAI criteria on pneumococcal polysaccharide response for diagnosis of SAD cannot be applied to bead-based assay results, and that a twofold increase is not attained in many healthy individuals for serotypes tested with ELISA. Instead, we propose to use method- and serotype-specific p5 cutoff values from a healthy population, as we have established with this study. This report confirms our earlier findings that the specificity of AHA for the detection of SAD is low. The established cutoff values will allow clinical validation of the *S. typhi* Vi assay and PPV response measured by bead-based assay to diagnose SAD in a patient population.

## Ethics Statement

This study was carried out in accordance with the recommendations of the Ethic Committee of the University Hospitals Leuven with written informed consent from all subjects. All subjects gave written informed consent in accordance with the Declaration of Helsinki. The protocol was approved by the Ethics Committee of the University Hospitals Leuven.

## Author Contributions

HS, BB, and IM designed the study, analyzed and interpreted the data, and drafted the manuscript. BK, CJ, M-PE, DD, and XB were responsible for parts of the data acquisition and have critically revised the manuscript. CJ, GF, and LM helped with data interpretation and revision of the manuscript. RS, MP, KB, MB, FV, NL, ID, and WP have contributed to recruitment of volunteers and data acquisition and have revised the manuscript critically.

## Conflict of Interest Statement

VaccZyme™ Anti-*S. typhi* Vi human IgG EIA kits were donated by The Binding Site Group Ltd., Birmingham, UK. There are no other financial or commercial relationships to declare.
