# Supplementary material for: Fifth Percentile Cutoff Values for Antipneumococcal Polysaccharide and Anti-Salmonella typhi Vi IgG Describe a Normal Polysaccharide Response
Source: Front Immunol. 2017 May 12;8:546. doi: 10.3389/fimmu.2017.00546 (PMC5427071; doi:10.3389/fimmu.2017.00546)
Supplement: Supplementary file 1 [file Data_Sheet_1.doc]

*Supplementary Material*

Fifth percentile cut-off values for anti-pneumococcal polysaccharide and anti-*Salmonella typhi* Vi IgG describe a normal polysaccharide response

Heidi Schaballie, Barbara Bosch, Rik Schrijvers, Marijke Proesmans, Kris De Boeck, Mieke Boon, François Vermeulen, Natalie Lorent, Doreen Dillaerts, Glynis Frans, Leen Moens, Inge Derdelinckx, Willy Peetermans, Bjørn Kantsø, Charlotte Sværke Jørgensen, Marie-Paule Emonds, Xavier Bossuyt, Isabelle Meyts*.

*Correspondence: Isabelle Meyts: [Isabelle.meyts@uzleuven.be](mailto:Isabelle.meyts@uzleuven.be)

1. **Supplementary Figures**

**FIGURE S1.** Dot plot demonstrating the distribution of age of the study population. Box shows 25^th^, 75^th^ percentile and median. The whiskers are at the 10^th^ and 90^th^ percentile.


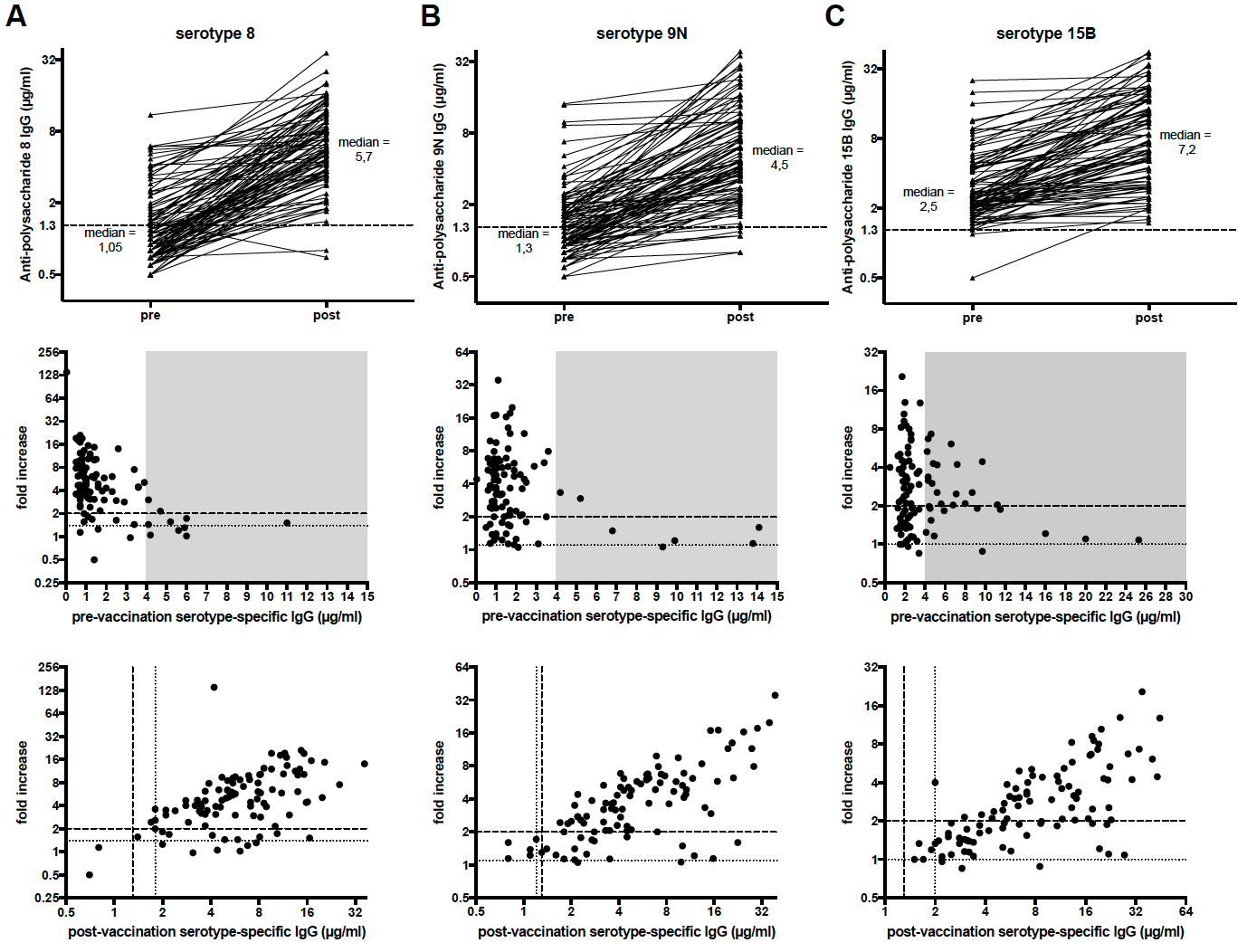


**FIGURE S2.** Serotype-specific paired pre- and post-vaccination IgG (upper graphs), fold increase over pre-vaccination IgG (middle graphs) and fold increase over post-vaccination IgG (lower graphs) for ELISA-tested serotypes 8 (A), 9N (B) and 15B (C). In the upper graphs, the 1.3 µg/ml cut-off for post-vaccination IgG, as used by the American Academy of Allergy, Asthma & Immunology (AAAAI), is indicated by a dashed line. According to the AAAAI criteria, the antibody response is considered normal, independent of post-vaccination IgG and fold-increase, when pre-vaccination IgG is greater than 4 µg/ml (grey area). In the middle graphs, the AAAAI threshold for fold increase of 2 is shown as a dashed line, the calculated 5^th^ percentile (p5) cut-off for fold increase in our study population is shown as a dotted line. The lower graphs show fold increase over post-vaccination IgG with dashed lines indicating AAAAI cut-offs and dotted lines indicating calculated p5 cut-offs in the study population.

**FIGURE S3.** Anti-*S. typhi* Vi IgG before and after vaccination with Typhim Vi vaccine in 100 paired serum samples from healthy subjects. Median pre- and post-vaccination levels are given. LLD: lower limit of detection of the ELISA (7.4 U/ml). Seven subjects had a pre-vaccination concentration greater than 50 U/ml.

**FIGURE S4.** Fold increase of anti-*S. typhi* Vi IgG against pre-vaccination anti-*S. typhi* Vi IgG levels. 95% prediction interval for fold increase is 2 (dotted line). Red dots are subjects with abnormal response according to our proposed cut-offs. At a pre-vaccination IgG level of ≥100 U/ml (grey area), Typhim Vi response is considered normal independent of post-vaccination IgG and fold increase.

**FIGURE S5.** Allohaemagglutinins in 100 healthy subjects. Boxes show 25^th^ to 75^th^ percentile and whiskers indicate 5^th^ to 95^th^ percentile. The p5 antibody titers are given right from the lower whisker.

1. **Supplementary Tables**

**TABLE S1.** Results and clinical history for the 15 healthy subjects with one or more responses abnormal.

| **Sex** | **Age** | **Interval blood samplings (days)** | **ELISA serotypes with good response (AAAAI cut offs) (x/3)** | **Total serotypes with response >p5 (x/15)** | **abnormal PPV response based on p5 cut offs** | **Pre-vaccination titer anti-Vi IgG (U/ml)** | **Post-vaccination titer anti-Vi IgG (U/ml)** | **Fold Increase anti-Vi IgG** | **abnormal Typhim Vi response** | **Blood group** | **Anti-A IgG (1/x)** | **Anti-A IgM (1/x)** | **Anti-B IgG (1/x)** | **Anti-B IgM (1/x)** | **abnormal AHA** | **Clinical history** | **Previous vaccination with Typhim Vi** |
| --- | --- | --- | --- | --- | --- | --- | --- | --- | --- | --- | --- | --- | --- | --- | --- | --- | --- |
| F | 22 | 26 | 0 | 6 | + | 3.7 | 43.5 | 11.8 | **-** | O | 256 | 128 | 4 | 8 | **-** | 1 pneumonia, 2-3 URTI/yr | 0 |
| F | 52 | 28 | 0 | 5 | + | 3.7 | 260.4 | 70.4 | **-** | A |  |  | 8 | 16 | - | / | 0 |
| M | 36 | 20 | 0 | 6 | + | 3.7 | 9.1 | 2.5 | + | A |  |  | 64 | 64 | - | 2-3 URTI/yr | 0 |
| M | 50 | 27 | 2 | 9 | + | 9.4 | 10.2 | 1.1 | + | A |  |  | 2 | 4 | - | 1 URTI/yr, 3 episodes of otorrhea | 2003 |
| M | 25 | 22 | 1 | 14 | **-** | 3.7 | 3.7 | 1.0 | + | A |  |  | 4 | 4 | **-** | / | 0 |
| M | 47 | 31 | 3 | 14 | **-** | 83.0 | 84.7 | 1.0 | + | B | 16 | 64 |  |  | **-** | 2-3 URTI/yr | 0 |
| F | 25 | 21 | 3 | 13 | **-** | 3.7 | 3.7 | 1.0 | + | O | 32 | 64 | 128 | 16 | **-** | / | 0 |
| M | 24 | 28 | 3 | 13 | **-** | 3.7 | 3.7 | 1.0 | + | A |  |  | 2 | 4 | **-** | 1 URTI/yr | 0 |
| M | 43 | 28 | 3 | 13 | **-** | 43.8 | 49.1 | 1.1 | + | O | 128 | 32 | 64 | 32 | **-** | 1 URTI/yr | 0 |
| F | 30 | 21 | 3 | 14 | **-** | 24.5 | 34.6 | 1.4 | + | O | 256 | 256 | 128 | 128 | **-** | 2-3 URTI/yr | 2004 |
| M | 24 | 28 | 3 | 15 | **-** | 186.9 | 600.0 | 3.2 | **-** | A |  |  | 1 | 2 | + | / | 2007 |
| F | 27 | 25 | 3 | 14 | **-** | 3.7 | 151.8 | 41.0 | **-** | A |  |  | 1 | 2 | + | 2-3 URTI/yr | 0 |
| M | 36 | 23 | 3 | 14 | **-** | 3.7 | 27.9 | 7.5 | **-** | O | 1 | 2 | 4 | 8 | + | 2-3 URTI/yr | 0 |
| F | 51 | 26 | 3 | 11 | **-** | 10.3 | 31.3 | 3.0 | **-** | O | 1 | 4 | 16 | 16 | + | / | 0 |
| F | 15 | 21 | 1 | 12 | **-** | 3.7 | 75.4 | 20.4 | **-** | A |  |  | 1 | 2 | + | / | 0 |

F: Female; M: Male; AAAAI: American Academy of Allergy, Asthma & Immunology; PPV: pneumococcal polysaccharide vaccine; AHA: allohaemagglutinins; URTI: upper respiratory tract infections; + indicates the presence of an abnormal response; - indicates a normal response.
